# Formatting the surgical management of Tessier cleft types 3 and 4

**DOI:** 10.4103/0970-0358.57192

**Published:** 2009-10

**Authors:** R. K. Mishra, Reetesh Purwar

**Affiliations:** Plastic Surgery Unit, Sushrut Institute of Plastic Surgery, 29, Shahmeena Road, Lucknow-3, U.P., India

**Keywords:** Tessier cleft types 3 and 4, Surgical formatting, Split approach

## Abstract

Tessier cleft types 3 and 4 are rare entities even among what are considered other rare craniofacial clefts. Very few cases have been reported worldwide, especially in the bilateral form. In the absence of any well-laid guidelines for management of such rare cases, plastic surgeons operate on such cases due to the inherent complexities in technique. To overcome this problem and provide a ground rule for surgical management of such cases, we propose an easier format with a ‘split approach’ of the affected areas. In our proposed formatting, we have divided the affected areas of the cleft into three components: 1. Lid component; 2. Lip component; and 3. Nasomalar component. Any person skilled in the plastic surgical art would appreciate that individual management of the aforesaid demarcated areas is easy as compared to the surgery of the entire craniofacial cleft, that too with the contemporary approach. We have evaluated this formatting technique with a ‘split approach’ in seven cases and found the results more convincing compared to those of classical methods. We invite the surgical fraternity to validate the surgical formatting in their settings and provide us with feedback on the same to consolidate these results.

## INTRODUCTION

Craniofacial dysrraphia, orbitomaxillary, and lateral facial clefts grouped under rare craniofacial clefts, are rare congenital anomalies in comparison to the more commonly seen cleft lip and palate.[1] The occurrence of rare craniofacial clefts is reported in 0.7–5.4 out of 1000 cases of cleft lip and palate.[2] The anatomical classification of these rare craniofacial clefts as proposed by Paul Tessier in 1976, is used even today for the identification and reporting of rare craniofacial clefts.[3] Tessier types 3, 4, and 5 clefts are rare with bilateral 3 and 4 clefts being even rarer. Although literature is replete with classification and morbid anatomy of such cases, surgical management of such cases is still a challenge. Furthermore, a major difficulty in understanding the management of these clefts arises from the fact that previous reports have focused on a single case or have grouped together different types of rare clefts, resulting in a lack of consensus about the management. Surgical management becomes more challenging as the classical surgical plan and markings used currently and described in literature are complex and confusing. There is also a dilemma regarding the proper mode in terms of time and stage of various surgical interventions. Therefore, in the absence of any well laid, practically adaptable guideline, the surgeon is either hesitant to attempt the surgery or dissatisfied with the ultimate postoperative results. This ultimately hampers the patients' interest. Hence, the need of the hour is an easy format for surgical management of these very rare and complex clefts.

The proposed ‘split approach’ to ease the plan of surgery assures a single-stage repair and provides better results in terms of aesthetics. We present here the management of seven cases of Tessier cleft types 3 and 4 in the unilateral and bilateral forms, specifically outlining the surgical management in detail with the aim to lay down certain ground rules towards planning and execution of surgery in these complex deformities.

## MATERIALS AND METHODS

In our prospective study, a series of seven patients with Tessier Cleft types 3 and 4, with age range 1.5 to 21 years were managed by our proposed surgical technique. Of these seven cases, two were males and five females; two of Tessier Cleft type 3 were bilateral whereas three out of the five cases of Tessier Cleft type 4 manifested as unilateral. The remaining two were bilateral forms. Cases were clinically examined and a CT scan was done to demonstrate the morbid/surgical anatomy. We deliberately omit the classification, detailed anatomy, and other usual detailed findings of these cases because this information is already present in the existing literature and extensively discussed. We mainly focus on our method of formatting and simplifying the surgical plan. We have used a ‘split approach’ to ease the surgical management of types 3 and 4 Tessier clefts. To simplify the classical surgical markings, we have divided the whole defect into three different segments and components, namely, *Lid, Lip,* and *Nasomalar components*. The lid and lip components are the same in types 3 and 4; it is only the nasomalar component that is different in types 3 and 4. We will discuss surgically relevant morbid or surgical anatomy further with example cases of each type (3 and 4) of Tessier cleft for the simplification of the proposed formatting. All cases have not been discussed in detail in this report for the sake of brevity but they do merit discussion in a separate article.

### Salient features in surgical anatomy with representative example

#### Tessier cleft type-3

A 12 year-old girl with bilateral Tessier cleft type-3 [Figure 1a] presented with an almost-absent premaxilla with scarred and almost-absent prolabial tissue (perhaps due to failed attempts of previous surgery). Medial incisors were missing but there was no clefting of the secondary palate. It is well known according to the classical description of Type-3 cleft by Paul Tessier, that the cleft starts as a general cleft of the lip at the vermilion, ascends upwards in the area of nasal ala (so the nasal ala and vestibule are usually absent or rudimentary) and through the lateral wall of the nose; it ends at the medial canthal area, medial to the inferior punctum. Southwards, the extension of this cleft is continued and noticed as a mild coloboma of the upper lid at the superior punctum area (representing the continuity of type-3 in the southward direction as Cleft type-11) as visible in [Figure 1a]. There was ectropion of the lower eyelid without any exposure keratitis, but with mild exposure stigmata of the conjunctiva. Vision was normal.

**Figure 1a:**
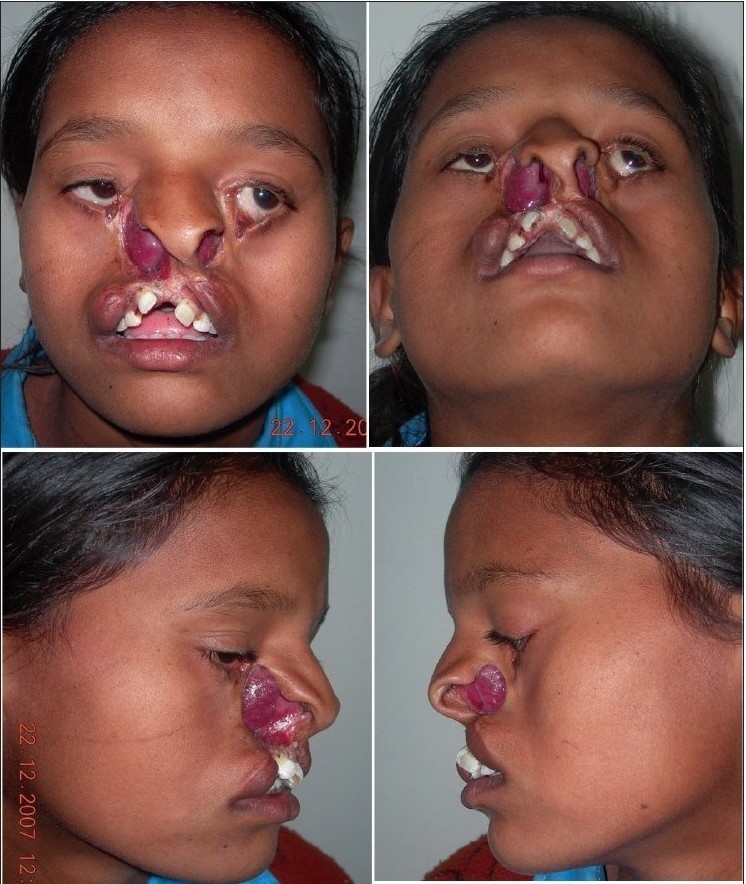
A 12 year-old girl with bilateral Tessier Cleft type-3

The second case of Tessier cleft type-3 revealed almost similar findings as the first one but in a milder form, except that whereas the premaxilla was normal, the prolabial tissue was rudimentary. CT scan findings confirmed the observation of surface deformities [Figure 1b]. The infero-medial wall and the rim of the orbit were slightly deficient but adequate enough to support the globe. Nasal bones were markedly hypoplastic, with only the nasal process of the frontal bone supporting the nasal dorsum.

**Figure 1b:**
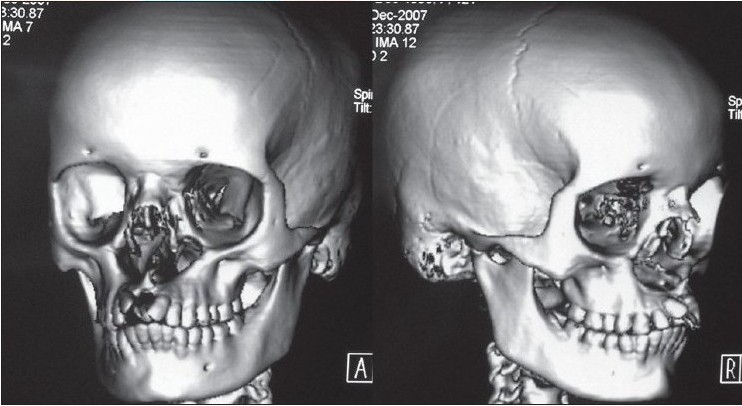
3-D CT scan of the same type-3 cleft patient showing severely hypoplastic nasal bone and maintained infra-orbital rim & orbital floor supporting the globe

#### Salient morbid anatomical features of Tessier cleft Type-3, critical to surgical judgement:

- As the cleft passes through the lateral part of nose, the alar and vestibular areas of the nose are almost absent or markedly hypoplastic and deficient. Therefore, strategies to address the construction of the lateral wall of the nose and nasal ala are critical.
- The cleft passes through the lateral wall of the nose where the critical confluence of the lacrimal apparatus is normally situated. Hence, the junction of the canaliculi, lacrimal sac, and the nasolacrimal duct is either grossly deficient or absent, and consequently beyond repair.

#### Tessier cleft type-4

A four year old male child with bilateral Tessier cleft type-4 [Figure 2a] presented with a cleft of the alveolus and primary palate with prominent and rotated premaxilla, the secondary palate having remained normal. Besides ectropion of the lower eyelid without exposure keratitis, there was abundant keratinization and severe exposure stigmata of exposed surrounding conjunctiva with significant mucosal and cutaneous pterygium. Vision was normal in this case. But in two other cases of wide clefts, the vision was lost as early as age of one year due to corneal opacity [Figure 2c]. Mucocele of the inferior canaliculi was present with mucous outpouring visible on exerting digital pressure. Midpenile hypospadias was noted. Computerized tomography revealed a complete separation of the premaxilla and nose from both maxillary segments, the pre-maxilla being attached through the nasal septum alone. There was no communication of the maxillary sinus with the nasal cavity (blind maxillary sinuses with no sign of pneumatization) (Figure 2b, right half). The infero-medial boundary of the orbit was hypoplastic with a guttering at the infero-medial portion of the inferior orbital rim. However, the major parts of the orbital floor and infraorbital rim (especially the middle and lateral) were adequately present to support the globe [Figure 2b, left half].

**Figure 2a:**
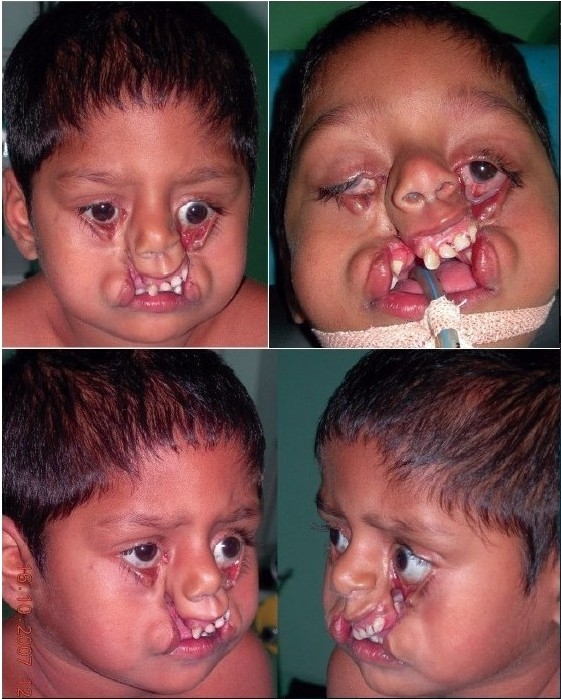
A four year-old boy with Tessier Cleft type-4

**Figure 2b:**
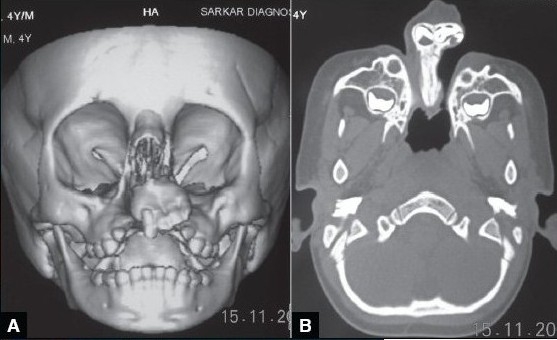
3-D CT scan of the same type-4 cleft patient with slightly deficient infra-orbital rim & orbital floor; it has a gutter type of appearance (A). There are separate & blind maxillary sinuses which never get a chance to unite with the nasal cavity (B)

In the other two cases of wide type-4, the cleft was so wide and the lateral segment was so displaced laterally that the orbital floor and infraorbital rim failed to provide any support to the globe. Hence, the normal upper eyelid failed to cover the cornea of the inferiorly displaced globe, leading to exposure keratitis and blindness [Figure 2c] as early as the 4^th^ day after birth [Figure 2d].

**Figure 2c:**
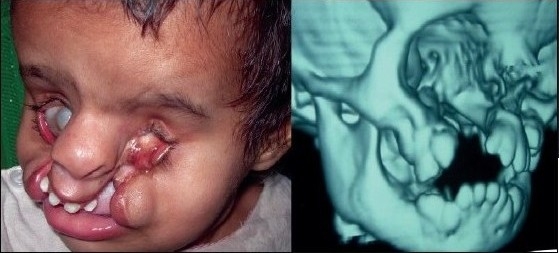
A case of bilateral Tessier cleft type-4 presented to us at one year of age. The infra-orbital margin & floor of the orbit were deficient (see CT Scan) & the globe was displaced inferiorly to such an extent that the upper eyelid was insufficient to cover the cornea. The cornea was already damaged

**Figure 2d:**
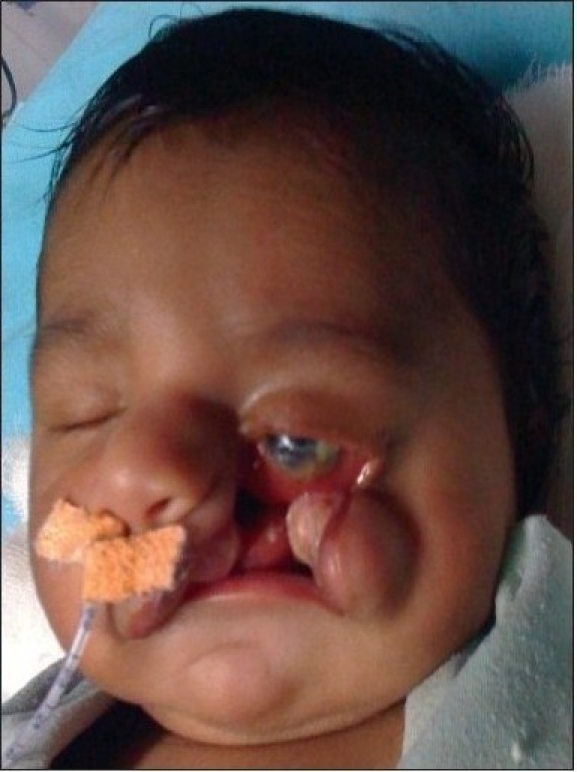
A case of unilateral Tessier cleft type-4 (wide) at the age of four days. The infra-orbital rim & floor of the orbit is almost completely deficient and is unable to support the globe, which is displaced so inferiorly that the cornea is seen to be already hazy. If no immediate precaution is taken (at least in the form of temporary tarsorrhaphy) to cover the cornea, exposure keratitis & blindness shall surely ensue (Photograph courtesy Dr. Jayachandran, Matha Hospital, Kerala)

As in the classical description of Type-4 cleft by Paul Tessier, the cleft starts lateral to the Cupid's bow (more lateral than type-3), ascends upwards lateral to the nose, ending in the medial canthal area, medial to the inferior punctum. Southwards, the extension of this cleft is continued and noticed as a mild coloboma of the upper lid at the medial third of the upper lid (representing the continuity of type-4 in the southward direction as Cleft type-10) [Figure 2a]. Spared in type-4 cleft, the nose appears rather small and pulled cranially; its air flow and breathing remaining unaffected.

#### Salient morbid anatomical features of Tessier cleft Type-4, critical to surgical judgement:

- The cleft, sparing the nose, passes laterally, not permitting the maxilla to unite with it. This leads to absence of pneumatization of the maxillary sinus (blind maxillary sinuses) [Figure 2b].
- The inferior punctum (critical in drainage of lacrimal secretion) is continued as a separate “*lateral lacrimal duct*” into this blind maxillary sinus. Due to clefting, this duct never gets a chance to unite with its medial counterpart or to drain into the lateral wall of nose [Figure 5b], thereby obstructing free flow of lacrimal secretions. This understanding of morbid anatomy is critical in surgically uniting this “*lateral lacrimal duct*” with the “*medial lacrimal duct,*” or directly implanting it into the lateral nasal wall to facilitate proper drainage of lacrimal secretion.
- Support to the globe inferiorly through the orbital floor and the ability of the upper lid to cover the cornea are very important when considering the timing of surgery. Early surgery to support the globe inferiorly is needed urgently in cases of wide type-4 clefts, especially in cases where the upper lid is unable to cover the cornea completely (clinical examination) and CT scan findings support the observation of lack of support to the globe due to a deficient orbital floor. Since early blindness was noted in two of our cases with wide type-4 case, we recommend that a temporary tarsorrhaphy must be performed to protect the cornea if definitive surgery is not possible due to medical / anaesthetic constraints [Figures 2c and 2d].

### Formatting of surgical plan (incision marking, dissection, and closure)

In order to simplify the surgical markings and subsequent management of types 3 and 4 Tessier clefts, we divided the whole defect into three different segments and components detailed as following: [Figures 3a and 3b]

**Figure 3a:**
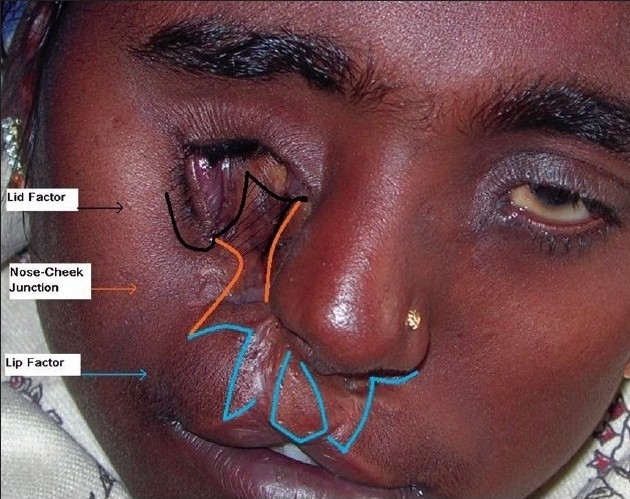
Simplified marking for Lid & Lip components. For lid component, the incision is marked at the junction of the lower lid & cheek as a back cut and pulled towards the medial canthal area for medial canthopexy. The shaded area of keratinized conjunctiva is to be excised. For Lip component, the incision is marked similar to Veau-III type lip repair with a back cut in the naso-labial crease, maintaining the height of the lip. See Figure 3c for the simplicity of the management of Lid & Lip components after medial canthopexy and Veau-III type lip repair

**Figure 3b:**
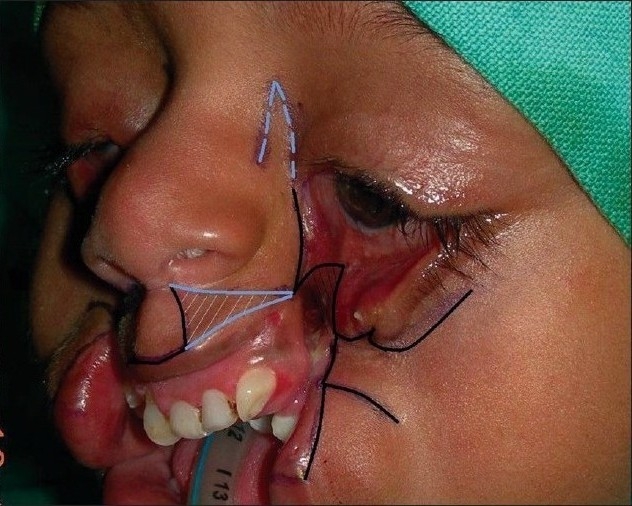
Incision markings described as in Figure 3a. Sometimes, following medial canthopexy, an inferiorly based transposition flap (dotted grey line) from high up in the lateral side of the nose may be required to fill the defect below the lower lid. The shaded area lateral to the philtral column is required to be excised

1. The ectropion of the lower eye lid (*Lid component*),
2. The cleft of the upper lip (*Lip component*), and
3. The gap between the nose and the malar area (*Nasomalar component*).

Management and markings of these three different sectors or areas are well known and form part of day-to-day work of every plastic surgeon. Our aim for this anatomical segregation of the overall surgery was to aid the cleft surgeon in managing these distinct areas individually without being overawed by complicated markings like in the management of types 3 and 4 cases *per se.* Moreover, surgeries for these three distinct areas could be combined to result in easily managed types 3 and 4 cases without the need to memorize the classical complicated markings.

#### The surgical steps (markings, dissection, and closure) of the Lid and Lip components are identical in types 3 and 4. It is the management of the nasomalar component that is different for types 3 or 4.

- Lid component: Markings preceeding surgical incision are made along the junction of the lower lid and the cheek (akin to a back cut) so as to elevate the medial end of the lower lid along with the inferior punctum up to the medial canthal area for medial canthopexy [Figures 3a and 3c]. At times, a flap from high up the lateral nasal wall or upper lid is needed to fill the defect below the lower lid after canthopexy [Figure 3b].
- Lip component: The prolabial segment and upper lip were marked as in Veau-III type B/L lip repair with a back cut in the nasolabial crease, maintaining the height of the lip. Vermilion turn-down flaps from the lateral lip element are required to from the prolabial vermillion [Figures 3a and 3c]. A triangular area of tissue needs to be sacrificed from the lateral side of the philtral marking in cleft type-4 [Figure 3b].
- Nasomalar component: A well placed Z-plasty is required to increase the distance between the ala and medial canthus, as in Tessier type-3 or direct closure after tailoring of tissue is needed as in type-4. Proper tailoring of the nasomalar component area is the key to the management of this component with the following caveats:

**Figure 3c:**
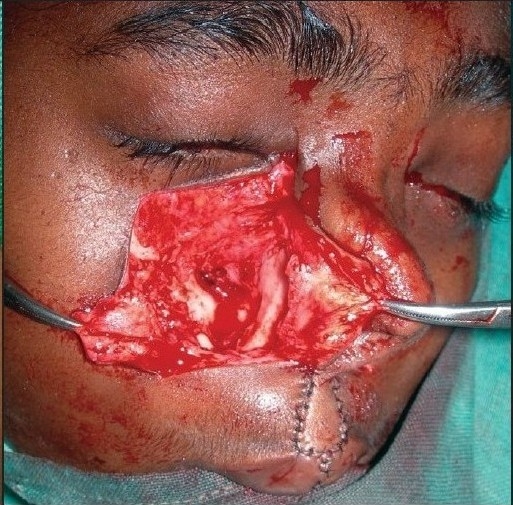
See the simplicity of the surgical steps of Lid & Lip components as after medial canthopexy and Veau-III type lip repair

**Figure 3d:**
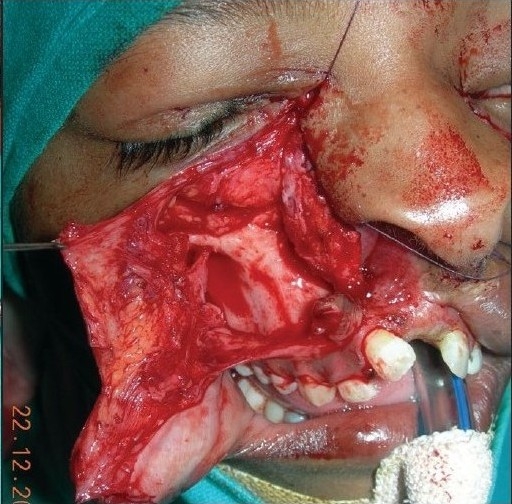
Plane & extent of dissection (subperiosteal and up to the lateral border of the maxilla & zygoma)

**In Type-3 clefts**, there is generally gross shortage of tissue in the nasal alar and vestibular areas, with exposed nasal lining and turbinates. The lining of the lateral nasal wall was created by a turning in of approximately 1 cm-wide flap from the cheek area skin and suturing it to a narrow turned-in flap of available alar tissue [Figures 4a and 4b] that is available. Due care should be taken so that almost all of the lining of the lateral nasal wall is created by this turned-in flap of cheek tissue, the turned-in tissue from the alar segment being minimal to facilitate coaptation. The remaining alar tissue should be meticulously saved at this stage for the creation of the deficient ala at a later date. Nasomalar tissue closure requires reconstruction by Z-plasty and creation of the ala through rotation and suturing of whatever alar tissue remains [Figures 4c and 4d].

**Figure 4a:**
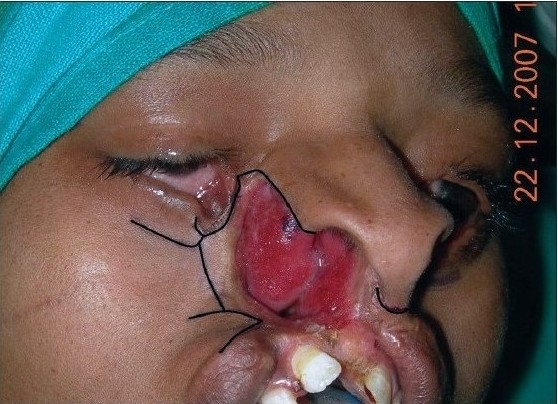
Markings for the management of the nasomalar component in case of Tessier cleft type-3. See most of the lateral wall of nose (lining) was created by the turn-in flap of cheek tissue which is stitched with a turn-in flap of the lateral nose & alar skin

**Figure 4b:**
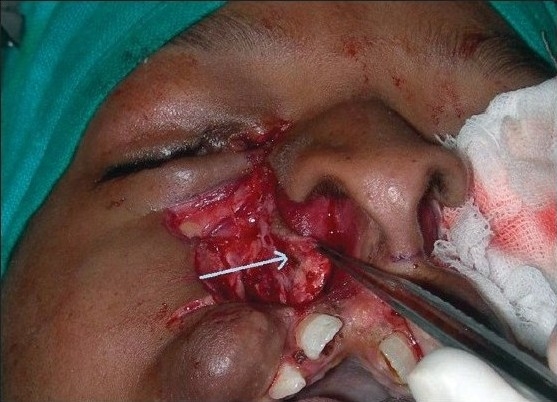
Management of nasomalar component in case of Tessier cleft type-3. See most of the lining of the lateral wall of the nose was created by the turn-in flap of cheek tissue which is stitched with a turn-in flap of the lateral nose & alar skin

**Figure 4c:**
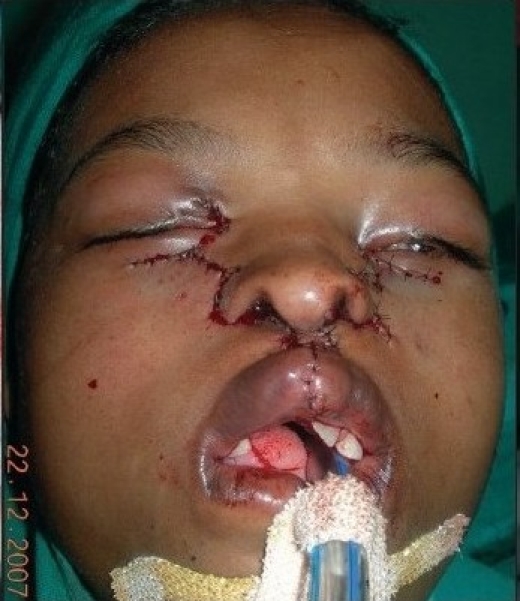
Closure of the nasomalar component in type-3 cleft after a Z-plasty. Ala was created by rotating & stitching whatever alar tissue was present (worm's eye view)

**Figure 4d:**
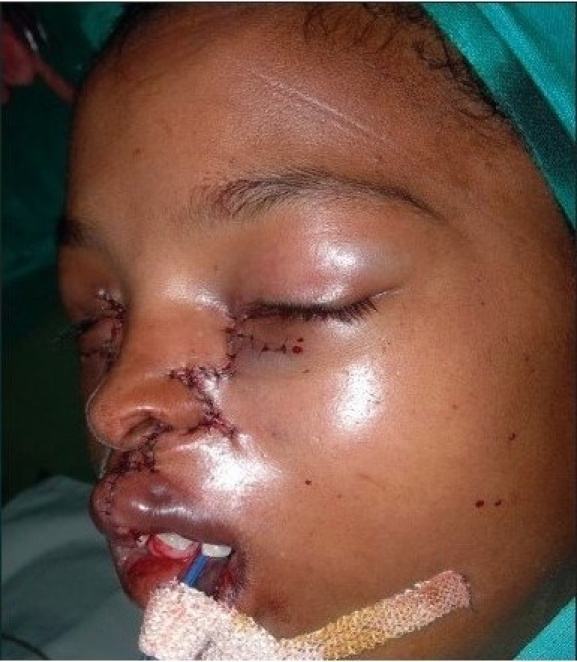
Closure of the nasomalar component in type-3 cleft after a Z-plasty (lateral view)

**In Type-4 cleft** the inferior canaliculi (starting from the inferior punctum, which is lateral to the cleft) ends in the blind maxillary sinus through a lateral duct without connecting to the nasal cavity. This “*lateral lacrimal duct*” was incised below and united to the “*medial lacrimal duct*” (starting from the superior punctum, which is medial to the cleft) that opens into the nose [Figure 5b]. (This step is not required/possible in Tessier cleft type-3 because the cleft, in that case, actually passes through this important area effacing the entire lacrimal system beyond recognition. In type-4 cleft cases, direct closure of the remaining defect is accomplished after adroit and ingenious tailoring. The portion of tissue infero-lateral to the nasal ala should be nimbly pulled infero-laterally so that it fits snugly into the triangle of the back cut in the naso-labial crease [Figures 5a, 5c, 5d, and 5e].

**Figure 5a:**
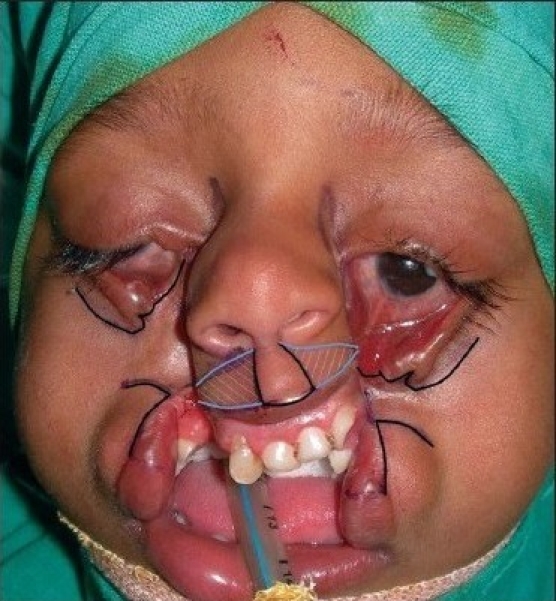
Marking (as described) in B/L Tessier cleft type-4. Shaded area in the upper lip shows zone of excision

**Figure 5b:**
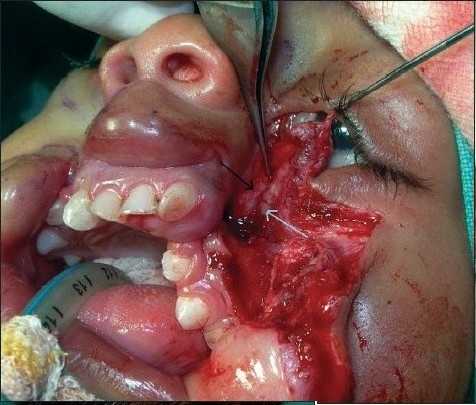
Dissection of nasomalar component in a case of B/L Tessier cleft type-4. Note the lateral lacrimal duct (going towards & ending in the blind maxillary sinus) and medial lacrimal duct going towards the lateral nasal wall

**Figure 5c:**
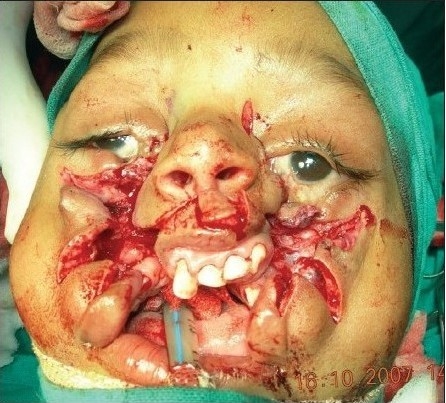
Dissection of B/L Tessier cleft type-4

**Figure 5d:**
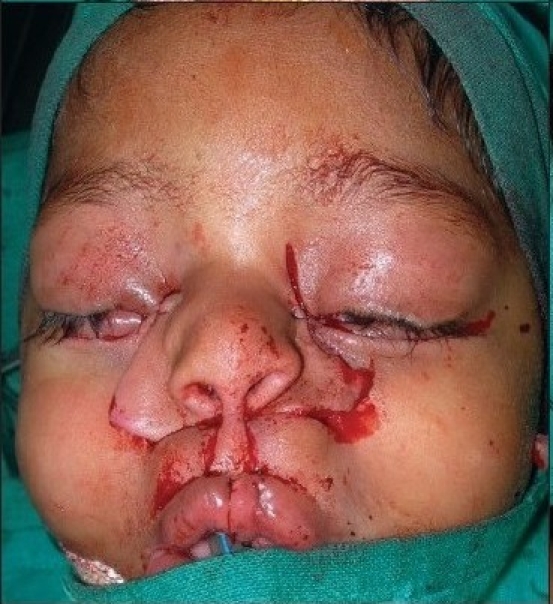
Repair of B/L Tessier cleft type-4 after medial canthopexy & upper lip repair and after application of key stitches

**Figure 5e:**
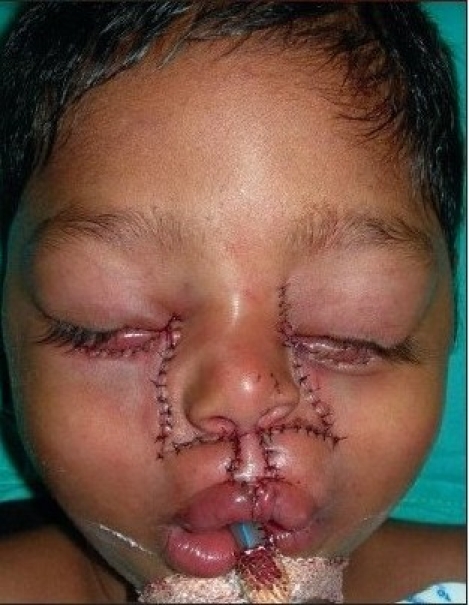
After final closure of B/L Tessier cleft type-4. There is usually no requirement of Z-plasty in the nasomalar area and the tissue from the lateral part of the nose naturally falls into the defect created by back-cut of the lip in the nasolabial crease. This tissue is pulled laterally and stitched snugly in the gap of the back-cut

All the dissections were done in the subperiosteal plane and laterally up to the lateral border of the maxilla and zygoma [Figure 3d]. As shown in the markings, all the incisions and the Z-plasties should be, through and through, bone-deep and three-dimensional up to the subperiosteal plane. This will amply mobilize the cheek tissue medially.

Suturing was performed in the following steps: 1. Medial canthopexy [Figure 3c]; 2. B/L Veau-III type closure of lip [Figure 3c], and 3. Tackling the nasomalar junction area [Figures 4c and 4d for Type-3 and Figures 5d and 5e for Type-4]. Closure must be done in three layers in the eyelid and upper lip area (lining, muscle/tendon, and skin) and in two layers (muscle and skin) in the nasomalar area. Care should be taken during closure to ensure proper approximation and suturing of the muscular layer to facilitate tension-less and accurate approximation of the overlying skin.

## RESULTS

**Figure 6:**
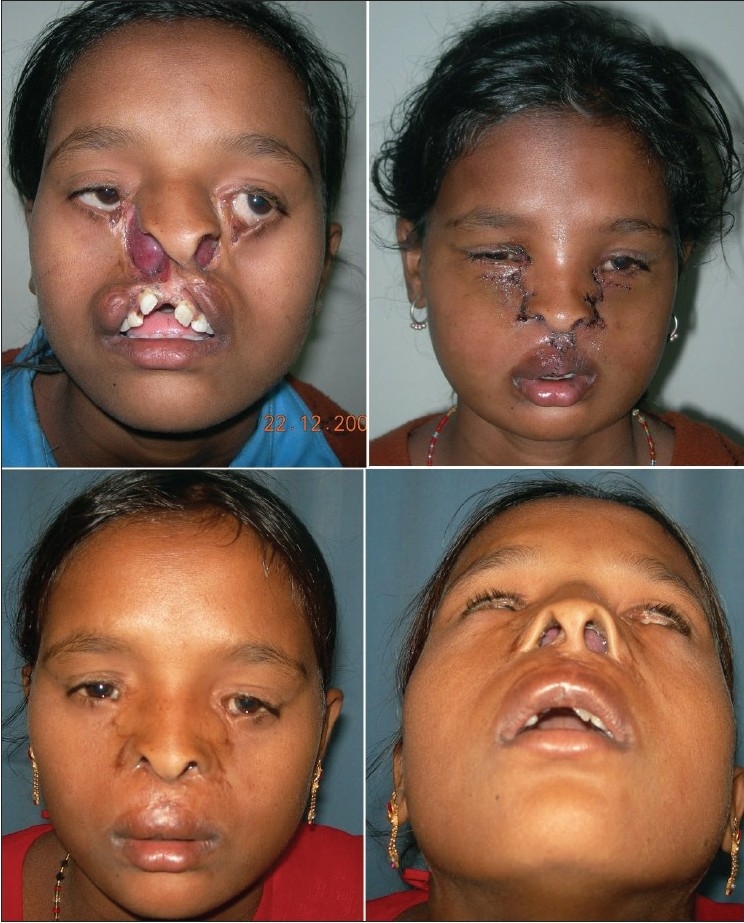
Pre- & postoperative photos of a case of B/L Tessier cleft type-3

**Figure 7:**
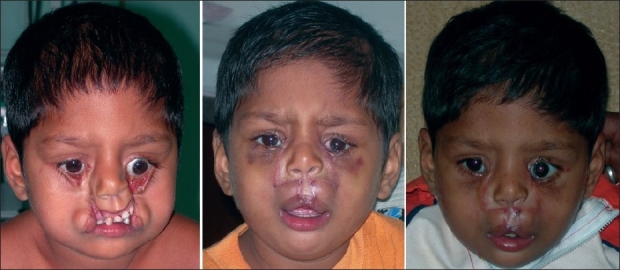
Pre- & postoperative photos of a case of B/L Tessier cleft type-4

All patients showed satisfactory postoperative results. [Figures 6, 7] Our proposed formatting for the surgical management provides substantial technical ease while promising pleasing surgical results. Though our assumption may appear subjective, cleft surgeons would agree that this segregation of surgery into three components through our “split approach” is simple, practical and adaptable when compared to the classical technique.

## DISCUSSION

Rare craniofacial clefts have been classified and accepted worldwide on the basis of Tessier's classification proposed in 1976.[3] This numerical system describes 16 different primary clefts, with additional possible combinations that can significantly raise the total number of potentially describable clefts. Among the rarest of the craniofacial cleft are the Tessier types 3 and 4 clefts, and especially their bilateral forms.[4]

The surgical management of such cases is a challenge, given the rare nature of these anomalies and a lack of standard of care guidelines.[5–10] Furthermore, the classical method of management involves complicated markings, which surgeons find difficult to memorize and can dissuade many a surgeon from attempting surgery. Also, surgeons are often faced with complexities like the ideal age for surgical intervention and methods to ensure minimal scars in these cases. In this article, we have tried to address these issues and have attempted to provide guidelines to manage such cases effectively on the basis of our experience of seven cases of Tessier cleft types 3 and 4 in their unilateral and bilateral forms. We propose a surgical format using which the surgeons can overcome their initial inhibition to attempt such surgeries and ultimately, further the patient's interest.

The first challenge due to which the surgeon hesitates attempting surgery in Tessier cleft types 3 and 4 can be ascribed to the complex nature of markings. Most of us need to consult a diagram.[11] Literature is replete with management guidelines of such cases and even various attempts to provide a better and clear classification of rare clefts.[12] The surgical protocols described in literature are varied and depend on individual centres, but the most widely accepted (hence, described in text books) are the Australian Craniofacial Unit Treatment Protocols.[13,14] This protocol suggests repair of the cleft lip and palate in the 1^st^ year of life and also suggests early intervention in the case of exposure keratitis, although exact time is not mentioned. They also proposed orthodontic intervention to expand the arch and speech therapy during the school-going years (4–10-years). The protocol also suggests definitive bone grafting in the orbital floor (even if the bone graft is applied in childhood), orthognathic surgery and rhinoplasty after the completion of facial growth. The protocol also propounds the role of tissue expansion of the cheek to accommodate the bone graft over the cheek and malar area, if needed. Tessier described some useful skin flaps to repair these clefts in 1990, but these flap markings usually seem complicated and difficult to remember.[14] Stricker *et al.* have opted for a cheek rotation flap including the lower eyelid in the flap in patients with extreme skin shortage.[15] Menard *et al.* also mentioned the use of tissue expanders under the cheek skin to facilitate tension-free closure.[16] However, the complexity and surgical challenge remain the same. It is also known that even though each of these cases poses a very unique surgical challenge, some general and special principles have to be followed in the completion of the complex treatment modalities. We have proposed a simplified and easy way of reconstruction whichshall enable every plastic surgeon to manage the rarest of rare Tessier types 3 and 4 clefts effectively. This technique has shown promising results in seven cases. Above all, it has eliminated the surgeon's hesitation in taking up such cases for surgery. Our methodology provides an easy solution to the difficult markings described in literature.

The proposed methodology is based on a ‘split approach’ of the affected areas of the cleft, dividing it into three parts: 1. Lid component; 2. Lip Component; 3. Nasomalar component. Any person skilled in plastic surgery would appreciate that individual management of the aforesaid demarcated areas is easier than surgery of the entire cleft. The lip component may be corrected as a simple B/L cleft lip using the Veau-III method of B/L lip repair that plastic surgeons often perform in their daily operative schedules. Furthermore, an eyelid ectropion-like picture may be managed by a back cut or incision in the eyelid and cheek junction and pulling the lower lid towards the medial canthal area for medial canthopexy. Finally, the third component of the demarcated area, the nasomalar junction, has to be corrected as described above in the formatting of our surgical plan. This demarcation of the affected portion in three areas, plus some specific points related to that specific deformity or morbid anatomy, is easily understood and managed by any plastic surgeon with avarage skill. Thus, this ‘split approach’ can be used to correct the areas individually and put the final result in the form of well managed types 3 and 4 clefts. This also helps surgeons to get the consistent results without remembering the difficult markings. Basically, this technique helps the surgeon to overcome that initial inhibition associated with remembering complex drawings as described in literature, focusing only on surgery, thus providing better results.

The second challenge before the surgeon is the management of operative and postoperative stage/conditions of the patient. It is well known that cases with rare cranio-facial clefts of any type, 0–15, have many associated medical problems and have to be managed from birth to maturity by a regular medical help including a cranio-maxillo-facial team, speech therapist, and psychiatrist. However, in cases of Tessier types 3 and 4, the initial challenge is also maintenance (prevention) of eyesight in the subject and possible management to retain/restore the lacrimal drainage. Danger to the eye (exposure keratitis and subsequent loss of vision) is the main reason prompting very early intervention, perhaps as early as 1–2 days of birth. The onus lies on the surgeon to plan and execute the surgery so that patient incurs minimum damage to the eyes.

From our experience, we recommend an early intervention to prevent blindness in cases where the cleft is so wide (wide type-4 cases) that the globe of eye is not supported inferiorly. In such cases, the eye sinks a little downward and the upper lid is unable to cover the cornea; subsequent exposure keratitis results in permanent loss of vision. The duration may be as early as within 2–4 days of birth as we have seen in two of our cases that presented to us at one year of age with complete and irreparable vision loss. One of our patients was brought on the 4^th^ day of birth with a very wide type-4 cleft with signs of exposure keratitis [Figure 2d]. Thus, the inability of the upper lid to cover the cornea (wide type-4 clefts with inadequate inferior support to globe) can be considered as a red flag sign. In such cases, if definitive management is to be delayed due to some medical or anaesthetic restraint, at least temporary tarsorrhaphy should be performed to cover and save the cornea from blindness. In narrow and incomplete type-4 clefts and in most cases of type-3 clefts, clefting is medial to the equator of the globe, the lateral element of the orbital floor usually affording it sufficient support. The upper lid is sufficient to at least cover the cornea (if not the complete conjunctiva) to prevent blindness. Definitive management may be delayed in such cases until the child is deemed to be fit. However, in our cases, the surgeries were delayed due to the late presentation of cases.

Since we obtained promising results with our ‘split technique’, we recommend it to all who seek to efficiently manage types 3 and 4 clefts. Further repetition of cases will corroborate our findings in diverse clinical settings as well. We invite cleft surgeons to use this formatting technique in their practice. This feedback shall help us in validating our hypothesis.

## CONCLUSIONS

The surgical plan is easy if we consider upper lip, lower eyelid, and nasomalar junction area in three distinct components.

In Type-3 clefts, the crux of the management of the nasomalar component lies in the creation of the lateral nasal lining by the cheek tissue and thereby saving most of the alar tissue for alar reconstruction.

In Type-4, the “lateral lacrimal duct” needs to be divided inferiorly and united with the “medial lacrimal duct” to facilitate drainage of lacrimal secretion.

To avoid blindness, early surgical intervention is recommended. Inability of the upper lid to cover the cornea is the deciding factor. If definitive management is to be delayed for some medical or anaesthetic reasons, at least a temporary tarsorrhaphy should be done to prevent exposure keratitis.

**Figure 8:**
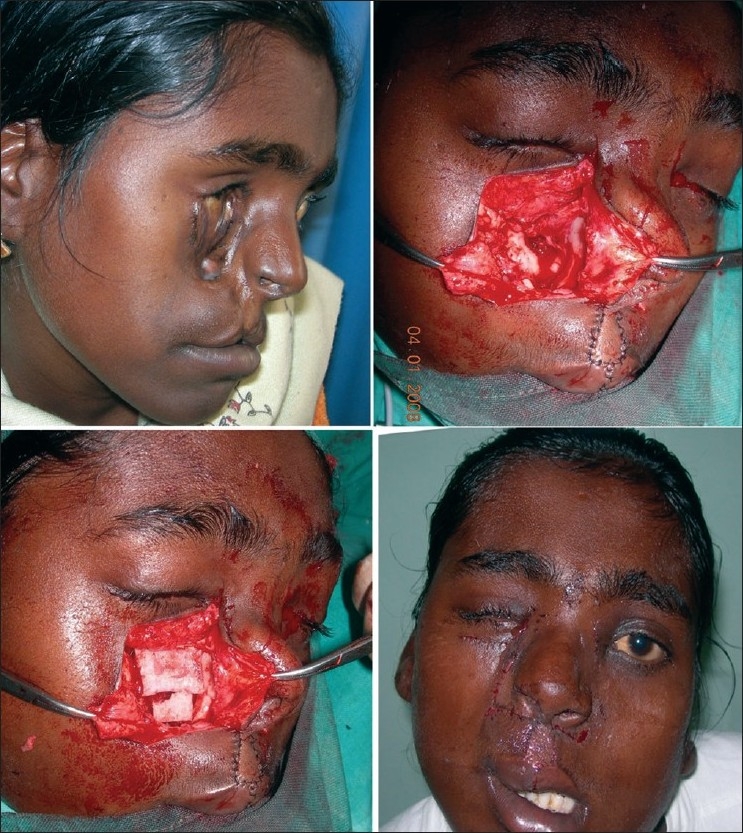
Adult case of U/L Tessier cleft type-4 with wide gap & depression in the nasomalar area required bone graft from the iliac crest

Bone graft is required in adult cases with wide bony defects and depression as shown in one of our cases [Figure 8].
